# Molecular characterization of extended-spectrum β-lactamases-producing *Escherichia coli* isolated from patients with bacteremia

**DOI:** 10.1007/s42770-026-02018-3

**Published:** 2026-07-13

**Authors:** Beatrice D. V. L. Souza, Guilherme Solveira, Guilherme F. R. de Souza, Henrique Orsi, Daiany R. P. De Lira, Iranildo do A. Fernandes, Adriano M. Ferreira, Alessandro L. Mondelli, Claudio T. Sacchi, Karoline R. Campos, Marlon B. N. Santos, Carlos H. Camargo, Rodrigo T. Hernandes

**Affiliations:** 1 Instituto de Biociências, Universidade Estadual Paulista (UNESP), Botucatu, SP Brazil; 2 Faculdade de Medicina, Universidade Estadual Paulista (UNESP), Botucatu, SP Brazil; 3 Instituto Adolfo Lutz, São Paulo, SP Brazil; 4 Departamento de Genética, Microbiologia e Imunologia, Instituto de Biociências, Universidade Estadual Paulista (UNESP), Câmpus de Botucatu. Rua Dr. Plinio Pinto e Silva. S/N. Distrito de Rubião Júnior,, Botucatu, SP 18618-691 Brazil

**Keywords:** Virulence factors, ExPEC, ESBL and bacteremia

## Abstract

Bacteremia is a major health problem that affects millions of people worldwide. *E. coli* is one of the main pathogens associated with bacteremia and its resistance to the antimicrobial drugs, including β-lactams, has significantly increased in recent years. In view of the above, our objective was to determine the antimicrobial resistance profile of 60 *E. coli* isolates obtained from patients with bacteremia, as well as to characterize extended-spectrum β-lactamases (ESBL) -producing isolates. The highest resistance rates were observed for ciprofloxacin (33.3%), levofloxacin (31.7%), cefuroxime (20%) and cefotaxime (20%). Furthermore, 12 isolates (20%) were ESBL producers. Genomic analysis showed that 58.3% of these ESBL-producing isolates belonged to the phylogroup B2, ST131, serotype O25:H4 and carried the following ESBL-encoding genes: *bla*_*CTX−M−15*_ (66.7%), *bla*_*CTX−M−8*_ (16.7%), *bla*_*CTX−M−2*_ (8.7%) or *bla*_*CTX−M−27*_ (8.7%). Our data highlight the high frequency of *E. coli* ST131 among the ESBL-producing isolates obtained from patients with bacteremia in Botucatu, Brazil.

**Supplementary Information:**

The online version contains supplementary material available at 10.1007/s42770-026-02018-3.

Bacteremia is a major health issue that affects millions of people worldwide and is characterized by the presence of bacteria in the bloodstream [1, 2]. The incidence increases progressively with advancing age, ranging from 110 cases per 100,000 people per year among adults aged 55 to 75, to 319 cases per 100,000 among those over 80 years old. *Escherichia coli *is one of the main etiological agents of bacteremia, most frequently originating from the urinary tract [3].

Sepsis is a life-threatening syndrome resulting from a dysregulated inflammatory response, often triggered by bacteremia, leading to tissue and organ damage, long-term health complications in survivors, and is among the leading causes of death worldwide [4, 5]. The incidence of sepsis has been reported to be higher in females, whereas mortality rates are higher in males. Interestingly, both the incidence and mortality of sepsis are highest in early childhood and in old age, and lower in the intermediate age groups [6].

*E. coli* that causes infections in extraintestinal sites are termed extraintestinal pathogenic *E. coli *(ExPEC) [7]. Several virulence factors contribute to ExPEC pathogenicity, such as adhesins, invasins, iron acquisition systems, protectins and toxins [8–10]. Adhesins are responsible for mediating bacterial adherence to host cells. For example, type 1 pili binds to mannose-containing glycoproteins on the surface of host cells, enabling the bacteria to attach to various cells, including human bladder epithelial cells [11, 12]. Similarly, the type P fimbriae bind to different glycolipids on host cells surfaces and is highly associated with cases of acute pyelonephritis [13, 14]. Moreover, there are also adhesins from the Afa/Dr family, which stimulate receptor clustering, and lead to functional and structural cell injury [15]. Another important virulence factor found in some ExPEC isolates is the IbeA (brain microvascular endothelial cells invasion) protein, that promotes invasion of brain endothelial cells by enabling the bacteria to pass the blood-brain barrier [16].

Iron acquisition systems enable the iron uptake from the host, which is essential for bacterial metabolism [17]. For iron acquisition, ExPEC isolates produce iron-chelating molecules called siderophores. Outer membrane receptors bind ferric-siderophore, which are transported into the periplasm and then across the inner membrane via ABC transporters. Iron is then released through multiple reactions and becomes available to the bacterial metabolism. Examples of siderophores include: aerobactin, yersiniabactin, and salmochelin [18].

Protectins provide bacteria with the ability to evade the host immune response system [10]. The majority of the ExPEC isolates produce group II type of capsules which is essential for protection from complement-mediated killing during bloodstream infections (BSIs) [19, 20]. Additionally, TraT is an outer membrane protein which inhibits the terminal pathway of the complement system, thus protecting the bacteria from the serum’s bactericidal activities [21, 22].

ExPEC can produce and secrete several exotoxins that can interfere or cause damage to several cell types in the human host. The necro toxin CNF1, for example, leads to reorganization of the cytoskeleton of host cells [23], while the secreted autotransporter toxin (Sat), an autotransporter serine protease, causes tissue damage by altering cell morphology and inducing apoptosis [24, 25]. Additionally, one of the most important ExPEC toxins is α-hemolysin (HlyA), which interacts with cell membrane generating pores that leads to the release of ferric ions and nutrients [26].

The rise of antimicrobial resistance (AMR) is a global health concern, with high levels observed in pathogens responsible for BSIs. Among the pathogens of interest are extended-spectrum β-lactamases (ESBL)-producing *E. coli *[27], which wide dissemination may be attributed to the fact that enzyme-mediated resistance-encoding genes are horizontally transferable [28]. The most prevalent ESBL genes come from the *bla*_*CTX−M*_family, which encodes cefotaximases, responsible for hydrolyzing cephalosporins such as cefotaxime [29].

Based on the increase of AMR rates observed in previously published articles from the literature [30], this study aimed to characterize a collection of 60 *E. coli* isolates obtained from patients with bacteremia regarding the presence of virulence factor-encoding genes, resistance profile, as well as to sequence and molecularly characterize the ESBL-producing *E. coli* isolates.

A total of 60 *E. coli *isolates were obtained from distinct blood samples of patients with bacteremia at the Hospital das Clínicas da Faculdade de Medicina de Botucatu (HCFMB), between 2018 and 2021. Clinical and demographic data, such as age, sex, comorbidities and other conditions of interest were provided anonymously by the hospital’s archives. The study was conducted in accordance with the Declaration of Helsinki, being evaluated and approved by the Botucatu Medical School Ethical Committee for human experimentation (CAAE: 64315522.3.0000.5411). The type of infection was established as community-acquired when it was either present prior to hospital admission or incubating during admission, those acquired after admission were established as hospital-acquired infections [31].

The presence of several virulence factor-encoding genes was investigated by DNA amplification. A total of 22 genes encoding adhesins, toxins, protectins, and iron acquisition systems were selected based on the well-established roles of their corresponding virulence factors in the pathogenesis of extraintestinal infections, particularly BSIs [8, 10]. The Polymerase Chain Reaction (PCR) was performed using 7.5 µL of GoTaq Green Master Mix (Promega, Madison, WI, USA), with 5.5 µL of nuclease-free H_2_O, 1 µL of template DNA and 0.34 µM of each of the primers. The sequences and PCR assay conditions used in this study are described in the references cited in the Table S1. The visualization was performed through the GeneSnap (Syngene, UK) software, using the inGenius LHR gel documentation imaging system (Syngene, UK).

The antimicrobial resistance profile was investigated using the disk-diffusion method [32], performed in Mueller-Hinton agar (Bio-Rad Laboratories, France), with several antimicrobial drugs. Double Disk Susceptibility Test (DDST) was also performed to assess Extended-Spectrum Beta-Lactamase (ESBL) production, according to Clinical and Laboratory Standards Institute [33] criteria. The antimicrobial disks were acquired from Cefar Diagnóstica, São Paulo, Brazil.

The DNA from the 12 ESBL-producing *E. coli* isolates were extracted using a commercial extraction kit (Promega Inc., United States), and the libraries were prepared using the Nextera Kit (Illumina, United States). The genomes were sequenced by Illumina paired-end short-reads 2 × 150 using the NextSeq Sequencing System (Illumina, United States). Prior to assembly, raw reads were quality-checked using FastQC (v0.12.0) (https://www.bioinformatics.babraham.ac.uk/projects/fastqc/) [34]and screened for potential contamination using Kraken2 (v2.17.1) [35]via the Galaxy Europe platform [36, 37]. The draft genomes were assembled *de novo *using the CLC Genomics Workbench software (v16) (QIAGEN, Germany) and the metrics were assessed with QUAST (v5.3.0) [38]. Gene annotation was done using Prokka (v1.14.5) software (https://github.com/tseemann/prokka) [39].

First, the genome drafts were analyzed using the site ClermontTyping (v23.06) (http://clermontyping.iame-research.center/) [40], for phylogenetic group identification. Then, the online tools Multi Locus Sequence Typing (MLST) (v1.0.1) [41], SerotypeFinder (v2.0.1) [42], ResFinder (v4.7.2) [43–45], and FimTyper (v1.0) [43], available on Center for Genomic Epidemiology (CGE, https://www.genomicepidemiology.org/), were used for classification of the 12 ESBL-producing *E. coli* isolates sequenced into the distinct Sequence Types (STs) and serotypes, detection of the presence of resistance-encoding genes, as well as *fimH* subtyping, respectively.

The Single Nucleotide Polymorphisms (SNPs)-based maximum likelihood tree was constructed using the online tool CSI Phylogeny (v1.4) [46–50] also available on CGE, with the *E. coli* ATCC8739 (accession number: NC_010468.1) strain used as reference, and the visualization was carried out using the online tool Microreact (v293) (https://microreact.org/). For comparative purposes, the pan- and core-genome analysis of the 12 ESBL-producing *E. coli* isolates sequenced in this study was performed using the Roary software (v3.13.0) (https://github.com/sanger-pathogens/Roary) [51].

From 2018 to 2021, 33,522 blood cultures were performed at the Hospital das Clínicas da Faculdade de Medicina de Botucatu (HCFMB). A total of 6,904 blood cultures (20.6%) were positive, among which 445 (1.3%) were positive for *E. coli*. Of these, 60 *E. coli* isolates were randomly selected for the present study. Among the 60 patients from whom the *E. coli* isolates causing BSIs were obtained, the majority were female (56.7%, 34/60), and the most common age group was between 41 and 65 years (51.7%, 31/60). Furthermore, most patients acquired the BSIs in the community (61.7%, 37/60), and the most common outcome was discharge (71.7%, 43/60) (Table S2). Infections caused by ESBL-producing *E. coli* isolates did not have a statistically significant impact on the outcomes (hospital discharge or death) of patients with bacteremia included in this study (*P* > 0.05), as determined using a two-tailed Fisher’s exact test (https://www.graphpad.com/quickcalcs/contingency1/).

All 60 *E. coli* isolates showed at least one of the virulence factor-encoding genes investigated and the maximum found was 16. The most frequent virulence factor-encoding genes detected were: *fimH* (95%, 57/60), *sitA* (75%, 45/60), *irp2* (66.7%, 40/60), *ompT* (63.3%, 38/60), *kpsMTII* (56.7%, 34/60), and *traT* (50%, 30/60) (Figure S1). Moreover, we observed 45 distinct virulence profiles, and the most common were the virulence profiles numbers 12 (13.3%, 8/60, *fimH*), 15 (5%, 3/60, *fimH*, *papC*, *papA*, *sitA*, *irp2*, *iucD*, *iha*, *ompT*, *kpsMTII*, *traT*, *sat*, *hlyA* and *cnf1*), and 19 (5%, 3/60, *fimH*, *papA*, *sitA*, *irp2*, *iucD*, *iha*, *ompT*, *kpsMTII*, *sat* and *vat*) (Figure S1). Consistent with our findings, previous studies have shown that *fimH*, *sitA*, *irp2*, *ompT*, *kpsMTII* and *traT* are genes frequently detected among *E. coli* isolates associated with BSIs, and have pointed out the high heterogeneity of virulence profiles found in this group of pathogens, which has complicated the identification of a common virulence profile associated with *E. coli *causing this type of infection [8, 9, 52–54].

The antimicrobials that showed the highest resistance rates were ciprofloxacin (33.3%, 20/60), levofloxacin (31.7%, 19/60), cefuroxime (20%, 12/60), cefotaxime (20%, 12/60), ceftriaxone (18.3%, 11/60), and cefepime (15%, 9/60). No resistance was observed for piperacillin-tazobactam, amoxicillin-clavulanate, amikacin, ertapenem, imipenem or meropenem. The DDST performed with the 60 *E. coli* isolates demonstrated that 20% of them (12 isolates) were classified as ESBL-producers (Table S3 and Figure S2). Of note, the high resistance rates observed for third-generation cephalosporins among the 12 ESBL-producing *E. coli* isolates were consistent with the presence of *bla*_*CTX−M*_genes in all of them, since CTX-M enzymes currently represent the dominant ESBL family in Enterobacterales [29, 55]. Likewise, the high frequency of fluoroquinolone resistance is epidemiologically consistent with the dissemination of multidrug-resistant ExPEC lineages, particularly those associated with ST131, in which β-lactam and quinolone resistance traits commonly coexist [56]. In Brazil, this phenomenon has been linked to the successful expansion of ExPEC lineages, especially ST131-related clones, that frequently combine *bla*_*ESBL*_genes with plasmid-mediated quinolone resistance determinants and/or Quinolone Resistance-Determining Region (QRDR) mutations [57].

Among the 12 patients infected with ESBL-producing *E. coli* analyzed in this study, half were female. Moreover, 7 acquired the infection during hospitalization, while 5 had community-acquired infections. It is important to note that half of the outcomes resulted in discharge, whereas the other half of the outcomes resulted in death (Table 1).

**Table 1:** Molecular characteristics of the ESBL-producing *E. coli* isolates and clinical information of the patients with bacteremia from which the ESBL-producing isolates were obtained

*E*. *coli* Identification	Molecular characteristics of the isolates					Clinical information				
	Phylogroup	MLST ST	in silico Serotype	ESBL gene	*fimH* type	Age	Gender	Type of Infection	Outcome	Comorbidities
EC_15	A	44	O101:H4	*bla*_*CTX−M−8*_	*fimH54*	80	F	Community	Death	Kidney failure
EC_18	B2	131	O25:H4	*bla*_*CTX−M−15*_	*fimH30*	82	M	Hospital	Discharge	Severe asthma
EC_22	B1	155	O9/O160:H51	*bla*_*CTX−M−27*_	*fimH32*	16	F	Hospital	Death	Leukemia
EC_25	B2	131	O25:H4	*bla*_*CTX−M−15*_	*-*	69	F	Hospital	Death	Hypertension and hypothyroidism
EC_28	C	410	ONT: H9	*bla*_*CTX−M−15*_	*fimH24*	63	M	Hospital	Discharge	Cecal appendix adenocarcinoma
EC_31	B2	131	O25:H4	*bla*_*CTX−M−15*_	*fimH30*	64	F	Hospital	Death	Breast carcinoma
EC_33	B2	131	O25:H4	*bla*_*CTX−M−15*_	*fimH30*	0	F	Hospital	Death	Preterm
EC_42	B2	131	O25:H4	*bla*_*CTX−M−15*_	*fimH30*	60	M	Community	Discharge	Hypertension
EC_52	B2	131	O25:H4	*bla*_*CTX−M−15*_	*fimH30*	90	M	Community	Discharge	Multiple myeloma
EC_54	A	10	O9/O101:H9	*bla*_*CTX−M−15*_	*fimH54*	68	M	Community	Death	Alzheimer
EC_59	F	648	O1:H6	*bla*_*CTX−M−2*_	*fimH27*	57	F	Hospital	Discharge	Acute myeloid leukemia
EC_61	B2	131	O25:H4	*bla*_*CTX−M−8*_	*fimH30*	53	M	Community	Discharge	Kidney transplant

The information regarding the assembly characteristics of the genomes obtained in this study can be found in Table S4. Most of the ESBL-producing *E. coli *isolates sequenced in this study (58.34%, 7/12,) were identified as belonging to the phylogroup B2, ST131 and serotype O25:H4, which is one of the four most common clones found in the ExPEC group [58]. Moreover, ESBL-producing *E. coli* isolates belonging to other *E. coli* phylogroups (A, B1, C and F), STs (ST10, ST44, ST155, ST410 and ST648) and serotypes (O1:H6, O9/O101:H9, O9/O160:H51, O101:H4, and ONT:H9) were also identified in the present study (Table 1). Of the 12 ESBL-producing isolates sequenced, all but one assigned to phylogroup B2 harbored *fimH30*, while all isolates from phylogroup A carried *fimH54*. Additionally, isolates from phylogroups B1, C, and F harbored the *fimH32*, *fimH24*, and *fimH27* subtypes, respectively (Table 1). Of note, the phylogroup B2 has previously been identified as the most prevalent phylogroup among *E. coli *isolates causing bacteremia [8, 52]. This is consistent with the high prevalence of ST131, one of the most widespread STs associated with urinary tract infections and BSIs, as well as with both community- and hospital-acquired infections [59–64]. Consistent with our findings, different *fimH* subtypes have been associated with distinct *E. coli* genomic backgrounds, as observed for *fimH30*, which is largely associated with ST131 [65–67]. The high frequency of B2-ST131/O25:H4 among the ESBL-producing isolates is particularly relevant because this lineage has repeatedly been linked to the successful dissemination of CTX-M enzymes and multidrug-resistant ExPEC causing invasive human disease.

Comparative genomic analysis of the 12 ESBL-producing *E. coli* isolates sequenced in this study revealed a pan-genome of 9,180 genes, including 3,212 (35.0%) core and 5,968 (65.0%) accessory genes. Among the accessory genes, 471 genes (5.1% of the pan-genome) were exclusively present in all *E. coli* isolates belonging to ST131 (Figure S3 and Table S5).The ESBL-encoding genes detected were: *bla*_*CTX*−M−15_ (66.7%, 8/12), *bla*_*CTX*−M−8_ (16.7%, 2/12), *bla*_*CTX*−M−2_ (8.3%, 1/12) and *bla*_*CTX*−M−27_ (8.3%, 1/12). Importantly, these genes were detected alongside multiple other resistance-encoding genes conferring resistance to different classes of antimicrobial agents, in addition to mutations in the quinolone resistance-determining regions (QRDR), as shown in Fig. 1 and Table S6. Importantly, the predominance of *bla*_CTX−M−15_ in our isolates, together with the detection of *bla*_CTX−M−8_, reinforces the association of bloodstream ExPEC with successful multidrug-resistant lineages. In Brazil, these ESBL variants have been identified within internationally disseminated clones, including ST131-related backgrounds, supporting the interpretation that the isolates described here belong to a broader regional epidemiological scenario [57, 68]. In bloodstream infections, this convergence of ESBL production and fluoroquinolone resistance is especially concerning because it narrows therapeutic options precisely in a clinical syndrome in which prompt administration of effective antimicrobial therapy is critical [69].

**Fig. 1:**
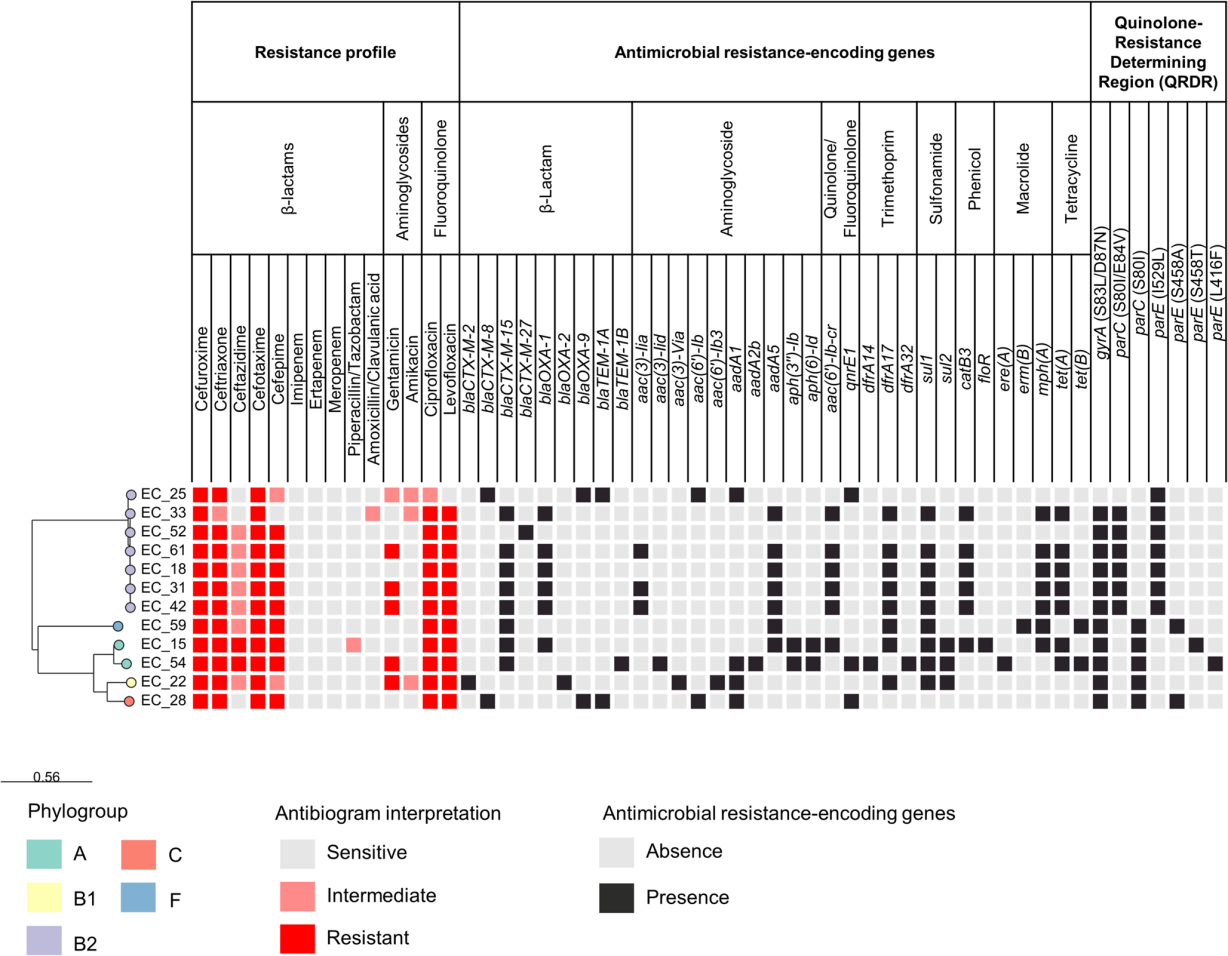
Resistance profile, antimicrobial resistance-encoding genes and quinolone resistance-determining regions (QRDR) identified in the Extended-spectrum β-lactamase (ESBL)-producing *E. coli* isolates evaluated in the present study. The ESBL-producing *E. coli* isolates studied harbored the following ESBL-encoding genes: *bla*_*CTX*−M−15_ (66.7%, 8/12), *bla*_*CTX*−M−8_ (16.7%, 2/12), *bla*_*CTX*−M−2_ (8.3%, 1/12) and *bla*_*CTX*−M−27_ (8.3%, 1/12)

This study demonstrated that *E. coli* isolates causing BSIs carry multiple virulence genes, with high prevalence of *fimH*, *sitA*, and *irp2*. Furthermore, ESBL production was observed in 20% of isolates, mainly associated with the presence of *bla*_*CTX−M−15*_ in the high-risk ST131 clone. The coexistence of virulence and resistance genes underscores the threat posed by ExPEC and highlights the need for continuous genomic surveillance.

## Supplementary Information

Below is the link to the electronic supplementary material.

Supplementary Material 1 (XLSX 1.21 MB)

Supplementary Material 2 (DOCX 152 KB)

## Data Availability

All data generated in this study are presented in the Tables and Figures included in the main manuscript and supplementary material. The GenBank accession numbers for the genome assemblies generated in the present study are listed in Table S4 and can be accessed at https://www.ncbi.nlm.nih.gov/.
