# Supplementary material for: Molecular characterization of extended-spectrum β-lactamases-producing Escherichia coli isolated from patients with bacteremia
Source: Braz J Microbiol. 2026 Jul 13;57(1):202. doi: 10.1007/s42770-026-02018-3 (PMC13365293; doi:10.1007/s42770-026-02018-3)
Supplement: Supplementary file 1 — Supplementary Material 1 (XLSX 1.21 MB) [file 42770_2026_2018_MOESM1_ESM.docx]

**Figure S1. Virulence profile of the 60 *E. coli* isolates studied.** A total of 45 distinct virulence profiles were identified. The most frequent were profile 12 (13.3%), profiles 15 and 19 (5% each), and profiles 3, 4, 7, and 21 (3.3% each). The remaining 38 profiles were observed only once (1.7%). The *sfa/focDE* gene was not detected in any of the *E. coli* isolates studied.

**Figure S2. Resistance profile of the 60 *E. coli* isolates studied.** Notably, 12 (20%) isolates were classified as ESBL-producers.


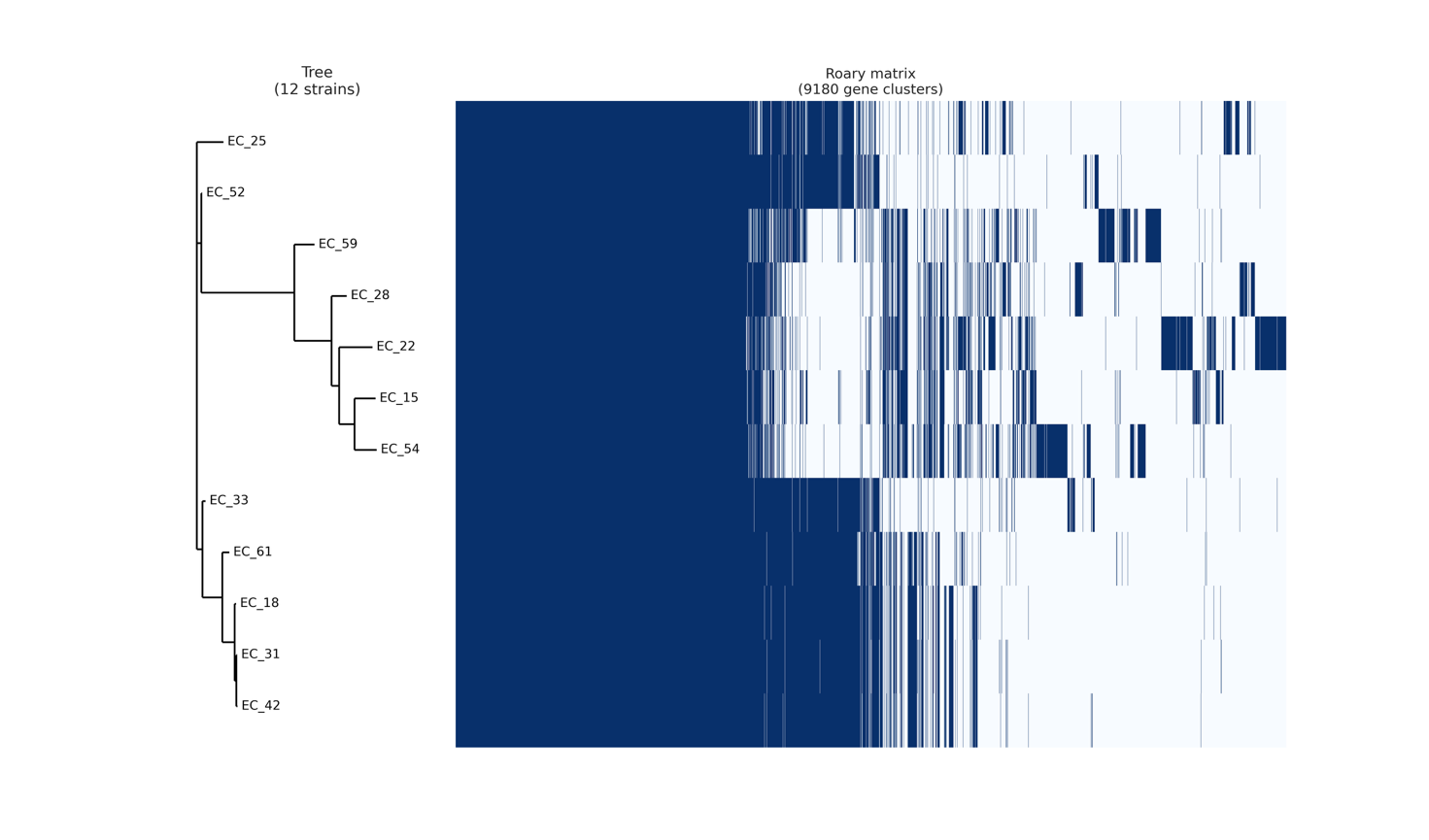


**Figure S3. Pan- and core-genome analysis of the *E. coli* isolates associated with bloodstream infections sequenced in this study.** The figure shows the presence (blue) or absence (white) of gene clusters (vertical axis) across the 12 genomes analyzed (horizontal axis). Isolates were grouped according to hierarchical clustering based on gene presence–absence patterns. The pan-genome comprised 9,180 genes, including 3,212 (35.0%) core genes and 5,968 (65.0%) accessory genes. Among the accessory genes, 471 (5.1% of the pan-genome) were exclusively present in all ST131 isolates.
